# Short-term and long-term outcomes of robotic rectal surgery—from the real word data of 1145 consecutive cases in China

**DOI:** 10.1007/s00464-019-07170-6

**Published:** 2019-10-10

**Authors:** Wenju Chang, Ye Wei, Li Ren, Mi Jian, Yijiao Chen, Jingwen Chen, Tianyu Liu, Wenbai Huang, Shangjin Peng, Jianmin Xu

**Affiliations:** 1 Colorectal Cancer Center; Department of General Surgery; Zhongshan Hospital, Fudan University, 180 Fenglin Road, Shanghai, China; 2 Shanghai Engineering Research Cancer of Colorectal Cancer Minimally Invasive (17DZ2252600), Shanghai, China; 3 Department of General Surgery, Jinshan Hospital, Fudan University, Shanghai, China

**Keywords:** Rectal cancer, Robotic rectal surgery, Sexual function, Urinary function, Oncological outcome

## Abstract

**Background:**

Due to a limited patient sample size, substantial data on robotic rectal resection (RRR) is lacking. Here, we reported a large consecutive cases from the real word data to assess the safety and efficacy of RRR.

**Methods:**

From September 2010 to June 2017, a total of 1145 consecutive RRR procedures were performed in patients with stage I–IV disease. We conducted an analysis based on information from a prospectively designed database to evaluate surgical outcomes, urogenital function, and long-term oncological outcomes.

**Results:**

Of three types of RRR performed, 227 (24.2%) were abdominoperineal resections, 865 (75.5%) were anterior resections, and 3 (0.3%) were Hartmann. Conversion to an open procedure occurred in 5.9% of patients. The overall positive circumferential margin rate was 1.3%. Surgical complication rate and mortality were 16.2% and 0.8% within 30 days of surgery, respectively. Mean hospital stay after surgery and hospital cost were 6.3 ± 2.9 days and 10442.5 ± 3321.5 US dollars, respectively. Risk factors for surgical complications included male gender, tumor location (mid-low rectum), combined organ resection, and clinical T category (cT3–4). Urinary function and general sexual satisfaction decreased significantly 1 month after surgery for both sexes. Subsequently, both parameters increased progressively, and the values 1 year after surgery were comparable to those measured before surgery. At a median follow-up of 34.6 months, local recurrence and distant metastases occurred in 2.3% and 21.1% of patients, respectively.

**Conclusions:**

Robotic rectal resection was safe with preserved urogenital function and arrived equivalent oncological outcomes in a nonselected group of patients with rectal cancer.

A robotic approach with superior dexterity and precise movements of the robotic arms provides the surgeon with better exposure and greater ergonomic comfort during dissection of small anatomical structures [1, 2]. Robotic rectal resection (RRR) has been proven to be a valid option with a lower open conversion rate than that of conventional laparoscopy for patients with rectal cancer [3]. Furthermore, robotic total mesorectal excision may overcome some difficulties associated with conventional laparoscopic rectal resection (LRR) [4] primarily in patients with mid-low rectal cancer [5]. However, the benefit of robotic rectal resection for urogenital function protection and oncologic outcomes remains controversial due to the limited sample size in reported studies [6, 7].

Previous studies show LRR is associated with similar [8] or higher [9] rates of sexual and urinary dysfunction compared to open rectal resection (ORR). The incidence of urinary and sexual complications after RRR is still not well known. Limited data indicated robotic total mesorectal excision (TME) may allow for better preservation of urinary and sexual functions when compared with both ORR and LRR [10]. Furthermore, regarding oncological outcomes of RRR, based on evidence from a limited number of cases and initial experiences, substantive data are still lacking [11]. Few centers worldwide have the capacity to perform real world large-scale studies of RRR because of low-volume sample sizes. However, colorectal surgeons at Zhongshan Hospital were early adopters of RRR and have performed more than 1700 such procedures in a nonselected group of patients with rectal tumors.

The purpose of this study was to define the safety and function preservation of the robotic rectal surgery base on a real-world database from a single center over an 8-year period in China. The surgical complication and risk factors, sexual and urinary function, and long-term oncological outcomes are described in this study.

## Materials and methods

### Patients

From September 2010 to June 2017, a total of 1211 consecutive patients are slated to undergo robotic rectal resection (RRR) with the Da Vinci S or Da Vinci Si Robot Surgical System. Of those, the robotic procedure was canceled in 66 cases after laparoscopic exploration revealed severe abdominal adhesions and intraperitoneal tumor dissemination. Ultimately, data from 1145 patients who underwent RRR were analyzed for this study. Patients were admitted to the study regardless of sex, age, AJCC/UICC (American Joint Committee on Cancer/Union for International Cancer Control) stage, type of intervention performed, or history of previous abdominal surgery. Data from patient hospital records were prospectively collected in a predesigned Excel file. Other clinical data were collected from computerized and archived patient charts. Postoperative data as well as surgical complications (30-day morbidity and mortality), sexual function, urinary function, and long-term oncological outcomes were assessed during follow-up.

A positive circumferential resection margin was defined as ≤ 1 mm from the specimen surface to the primary tumor or any tumor deposit. [12] Dissection planes are registered according to the description by Quirke. [13] The postoperative complications were defined as adverse events that occurred within 30 days after surgery. Complications were diagnosed and categorized according to patients’ symptoms, with the aid of laboratory and radiological evaluation to confirm clinical suspicions. Grading of complications was scored based on the detailed tables of the Surgical Complications Severity Scoring System proposed by Mazeh et al. [14].

The study was approved by the Fudan University Ethics Committee, and all patients were asked to provide informed consent. Urinary and sexual dysfunctions affecting quality of life (QoL) were assessed by means of specific self-administered questionnaires in all patients undergoing robotic TME. For evaluating urinary tract symptoms and the impact on QoL, the International Consultation on Incontinence Male/Female Lower Urinary Tract Symptoms questionnaires (ICIQ-FLUTS and ICIQ-MLUTS) were used [15]. Each module uses a common question format. Most questions use 5-point Likert scales to assess the presence or absence of a symptom and its severity, followed by a scale to assess the associated degree of bother, which is measured by a visual analog scale. For assessing male sexual function, the International Index of Erectile Function (IIEF) questionnaire [16] was adopted, and for female sexual function, the Female Sexual Function Index (FSFI) questionnaire [17] was adopted. These are 15-item self-administered questionnaires that analyze five factors: erectile function (sexual function for female), orgasmic function, sexual desire, intercourse satisfaction, and overall satisfaction. All of these questionnaires can effectively assess urinary and sexual function after prostate cancer [18] and rectal surgery [19, 20] as reported previously.

### Robotic rectal resection

The single-docking technique with four or five ports was used as described in our previous studies [21]. We performed the high dissection and low ligation for lymph node dissection and preserved the left colic artery in most patients. The splenic flexure of the colon was not routinely mobilized, depending on the tension of the anastomosis. Once the sigmoid colon, mesocolon, entire rectum, and mesorectum were mobilized completely, anterior resection (AR) with the double-staple technique or abdominoperineal resection (APR) was performed accordingly.

We routinely used a standardized approach to prevent anastomotic leakage (AL) for mid-low rectal tumors, which included preserving the left colonic artery to improve the blood supply to the anastomosis. A transanal drainage tube was placed to reduce anastomotic tension in patients with robotic LAR. A diversion stoma was not routinely performed except in those patients at a substantial risk of AL. If the anastomosis was below the peritoneal reflex, the dissected pelvic peritoneum was sutured to avoid leakage of feces, which could cause intraperitoneal peritonitis following AL. At the same time, a double cannula was placed near the anastomosis to monitor the occurrence of AL. When AL occurred, the double cannula located near the anastomoses could be used to wash and drain the feces, promote anastomotic healing, and avoid a salvage ileostomy.

### Statistical analysis

Statistical analysis was conducted with IBM SPSS Statistics software version 19 (SPSS Inc., IBM, Chicago, IL, USA). Categorical variables were analyzed using the *χ*^2^ test, and continuous variables were analyzed using the Student *t* test. The nonparametric Kruskal–Wallis test was employed to compare the qualitative variables for urinary and sexual function analysis. One-way analysis of variance with least significant difference multiple comparisons was used for analysis of quantitative differences between multiple groups. A logistic regression was used for multivariate analysis. Overall survival was calculated using the Kaplan–Meier method. *P* values less than 0.05 were considered statistically significant.

## Results

### Perioperative and pathological data

The 1145 patients who underwent robotic rectal resections (RRR) are summarized in Table 1. Briefly, they included 714 (62.4%) males and 431 (37.6%) females. The median age and body mass index (BMI) were 63 years (range 24–91) and 23.1 kg/m^2^ (range 15.1–35.0), respectively. A total of 138 (12.1%) patients are considered to have American Society of Anesthesiologists scores (ASA) of III–IV, 231 (20.2%) patients had a history of abdominal surgery, and 516 (45.1%) patients had comorbidities.

**Table 1:** Perioperative data

Patients characteristics	Value (*N *= 1145); *n* (%)
Gender	
Male	714 (62.4%)
Female	431 (37.6%)
Age (years)	63 (24–91)
BMI (kg/m^2^)	23.1 (15.1–35.0)
ASA score	
I–II	1007 (87.9%)
III	138 (12.1%)
History of abdominal surgery	231 (20.2%)
Digestive system	136 (11.9%)
Gynecology	79 (6.9%)
Other	16 (1.4%)
Comorbidity	516 (45.1%)
Cardiovascular diseases	341 (29.8%)
Diabetes	142 (12.4%)
Cerebrovascular disease	33 (2.9%)
Tumor location	
Upper rectum (10–15 cm)	367 (32.1%)
Mid rectum (5–9.9 cm)	423 (36.9%)
Lower rectum (0–4.9 cm)	355 (31.0%)
Operation performed	
AR	365 (31.9%)
LAR	500 (43.6%)
APR	277 (24.2%)
Hartmann	3 (0.3%)
Preoperative RT or CRT^b^	181 (23.7%)
Postoperative chemotherapy or CRT^b^	496 (43.3%)
Operative time (min)^c^	166.8 ± 31.6 (106–720)
Estimated blood loss (mL)^c^	73.8 ± 30.5 (5–400)
Blood transfusions (patients)	16 (1.4%)
Conversion to open surgery^d^	68 (5.9%)
Diverting stoma^e^	3 (0.3%)
Combined organ resection	133 (11.6%)
Liver	90 (7.9%)
Gynecological organs	20 (1.7%)
Urinary organs	7 (0.6%)
Other	16 (1.4%)
Time of first flatus passage (day)^c^	1.6 ± 0.1 (1–11)
Time of liquid diet (day)^c^	2.1 ± 0.5 (1–27)
Time of remove urinary catheter (day)^c^	2.1 ± 0.3 (1–28)
Postoperative hospital stay (day)^c^	6.3 ± 2.9 (4–45)
Total hospital cost (US dollars)^c^	10442.5 ± 3321.5 (5624.3–62924.9)

*BMI* body mass index, *ASA* American Society of Anesthesiologists, *AR* anterior resection, *APR* abdominoperineal resection, *RT* radiotherapy, *CRT* chemoradiotherapy

^a^Patients with T4 or N2 disease accepted radiotherapy or chemoradiotherapy before operations. Among of 778 patients with mid-low rectal cancer, 181 (23.7%) patients accepted preoperative RT or CRT

^b^Total 406 patients with high risk of relapse accepted adjuvant chemoradiotherapy, and 90 patients with unresectable distant metastases accepted chemotherapy after operation

^c^Value expressed by Mean ± SD (range)

^d^Conversion to open surgery was analyzed in 1145 patients undergone robotic rectum resection. During operations, 66 cases canceled robotic procedure after laparoscopic exploration due to severe abdominal adhesions and intraperitoneal tumor dissemination. The adjusted conversion rate was 11.1% (134/1211) if these 66 patients were calculated

^e^Diverting stoma was analyzed in 365 patients undergone AR and 500 patients undergone LAR

Tumor locations are detailed in Table 2. There were 367 (32.1%) patients with upper rectal tumors, 423 (36.9%) with middle rectal tumors, and 355 (31.0%) with low rectal tumors. Anterior resection (AR), low anterior resection (LAR), and abdominoperineal resection (APR) procedures were performed in 365 (31.9%), 500 (43.6%), and 277 (24.2%) patients, respectively. The mean operative time and estimated blood loss were 166.8 ± 31.6 min (range 106–720) and 73.8 ± 30.5 mL (range 5–400), respectively. Overall blood transfusion events totaled 16 (1.4%) within 30 days of surgery (Due to improve preoperative serious anemia (hemoglobin < 70 g/L) in 14 patients, and 2 patients suffered from major bleeding after operation). A diverting stoma was completed in 3 of 500 patients with LAR. The number of conversions to open procedures was 68 (5.9% conversion rate), of which, 60 cases were due to combined organ resection, and 8 for major bleeding or difficult tumor dissection. Following laparoscopic exploration, robotic procedures were canceled for 66 patients with severe abdominal adhesions or intraperitoneal tumor dissemination. The adjusted conversion rate was 11.1% (134/1211) if these 66 patients were calculated. The 133 (11.6%) patients accepted for combined organ resections are summarized in Table 1. A total of 181(23.7%) patients with T4 or N2 mid-low rectal cancer accepted preoperative radiotherapy or chemoradiotherapy. After surgery, 406 patients at high risk of relapse accepted adjuvant chemoradiotherapy, another 90 patients with unrespectable distant metastases accepted chemotherapy/target therapy after the primary tumor resection. The mean time of liquid diet and first flatus passage after surgery were 1.6 ± 0.1 days and 2.1 ± 0.5 days, respectively. The mean hospital stay after surgery and total hospital cost were 6.3 ± 2.9 days and 10442.5 ± 3321.5 US dollars, respectively (Table 2).

**Table 2:** Pathological data

Patients characteristics	Value (*N *= 1145); *n* (%)
AJCC stage (pathologic)^a^	
Benign tumors	6 (0.5%)
I	247 (21.6%)
II	322 (28.2%)
III	391 (34.1%)
IV	179 (15.6%)
Pathological type^a^	
Adenocarcinoma	1038 (90.7%)
Mucinous	98 (8.5%)
Other	9 (0.8%)
Tumor size (cm)	
≤ 5	788 (68.8%)
> 5	357 (31.2%)
Differentiation^a^	
Well	208 (18.1%)
Moderate	820 (71.6%)
Poor	108 (9.5%)
Other	9 (0.8%)
No. of harvested lymph nodes^b^	17 ± 10.5 (5–54)
Vascular invasion	155 (13.5%)
Perineural Invasion	256 (22.4%)
Positive DRM	6 (0.5%)
Positive CRM	15 (1.3%)
Quality of mesorectum^c^	
Complete	706 (90.2%)
Near complete	71 (9.1%)
Incomplete	0 (0%)
Resection degree of primary tumor	
R0	1126 (98.4%)
R1	21 (1.8%)
Resection degree of both primary tumor and distant metastases^d^	
R0	1034 (90.3%)
R1	111 (9.7%)

*AJCC* stage indicates the American Joint Committee on Cancer TNM classification, *DRM* distal resection margin, *CRM* circumferential resection margin (a positive circumferential resection margin was defined as ≤ 1 mm from the specimen surface to the primary tumor or any tumor deposit)

^a^Total 1145 patients were analyzed, including 1139 patients with malignant tumor and 6 patients with benign tumors

^b^Value expressed by Mean ± SD (range)

^c^According to Quirkes’ criteria [13]. Quality of mesorectum in 777 patients undergone LAR or APR were analyzed

^d^Total 111 patients were not accepted radical resection due to unresectable distant metastases (*n* = 90) and R1 resection of primary tumor (*n* = 21)

The mean number of harvested lymph nodes was 17 ± 10.5 (range 5–54). The positive rates of circumferential margin (CRM) and distant margin (DRM) were 15 (1.3%) and 6 (0.5%), respectively. The surgical quality of mesorectal excision calculated in 777 patients who underwent LAR or APR, according to Quirkes’ criteria, with “complete” in 706 (90.2%) patients and “near complete” in 71 (9.1%). No case was the mesorectum defined as “incomplete” by the pathologist. The pathological data are summarized in Table 3.

**Table 3:** Surgical complications

Characteristics	Value (*N* = 1145); *n* (%)
Total complications rate^a^	187 (16.3%)
Grade 1–2	159 (13.8%)
Grade 3	23 (2.0%)
Grade 4	4 (0.4%)
Grade 5	1 (0.1%)
Complications	
Infection events^b^	42 (3.7%)
Anastomosis leakage^c^	36/865 (4.2%)
AR	3/365 (0.8%)
LAR	33/500 (6.6%)
Urinary retention	28 (2.5%)
Blood transfusion^d^	16 (1.4%)
Ileus	15 (1.3%)
Organ dysfunction^e^	14 (1.2%)
Chyle leak	8 (0.7%)
Gastric motility disorders	7 (0.6%)
Thrombotic events	7 (0.6%)
Postoperative bleeding	6 (0.5%)
Others	7 (0.6%)
Mortality^a^	1 (0.1%)
Rehospitalization rate^f^	26 (2.3%)
Reoperation rate^f^	9 (0.8%)

*AR* anterior resection, *LAR* low anterior resection

^a^Surgical complication rate and mortality was analyzed within 30 days of operation following Mazeh system [14]. One patient died of hepatic failure after simultaneous hepatectomy for liver metastases

^b^Infection events included intraabdominal infection or abscess, catheter-derived infection, wound infection and lung infection, but excluded anastomotic leakage events

^c^Anastomosis leakage was analyzed in 365 patients who underwent AR and 500 patients who underwent LAR

^d^Due to improve preoperative serious anemia (hemoglobin < 70 g/L) in 14 patients, 2 patients suffered from major bleeding after operation

^e^Organ dysfunction included dysfunction of heart, brain, lung, liver, and kidney

^f^The rates of rehospitalization and reoperation which related to surgical complications were analyzed within 90 days of operation

### Surgical complication and risk factors

The number of overall surgical complications was 187 (16.3%). Grade 1 and grade 2 complications together accounted for 13.8%, while grade 3 and grade 4 complications were 2.0% and 0.4%, respectively. One patient died of hepatic failure after simultaneous hepatectomy for liver metastases within 30 days of the operation. The rehospitalization rate and reoperation rate associated with surgical complications within 90 days of surgery were 26 (2.3%) and 9 (0.8%), respectively.

We evaluated risk factors for surgical complications associated with RRR using a multivariate model, including factors that were statistically significant (*P *< 0.05) in a univariate analysis (Table 4). The male gender, tumors located at the mid-low rectum, combined organ resection, and clinical T category (cT3-4) were confirmed as the independent risk factors for surgical complications by multivariate analysis.

**Table 4:** Univariate and multivariate analyses of risk factors associated with surgical complications

Factor	Univariate analysis			Multivariate analysis		
	Odds ratio	95% CI	*P* value	Odds ratio	95% CI	*P* value*
Gender (male)	1.57	1.22–2.11	0.01	1.756	1.24–2.48	0.004
Age (≥ 70 years)	1.13	0.75–1.95	0.54			
CEA (≥ 5 ng/mL)	1.02	0.68–1.50	0.89			
CA199 (≥ 16.9 ng/mL)	1.09	0.70–1.84	0.69			
Neoadjuvant therapy (yes)	0.81	0.42–1.86	0.54			
BMI (≥ 25 kg/m^2^)	1.28	0.90–1.27	0.16			
History of abdominal surgery (yes)	1.14	0.75–1.32	0.53			
Comorbidity (yes)	1.30	0.56–3.50	0.53			
Operative time (≥ 240 min)	0.72	0.36–1.51	0.36			
Tumor location (mid-low rectum)	2.19	1.41–3.18	0.00	2.24	1.41–3.37	0.000
Combine organ resection (yes)	2.10	1.25–3.33	0.00	2.08	1.53–2.92	0.001
Estimated blood loss (≥ 100 mL)	1.50	0.86–2.69	0.15			
Differentiation (poor)	0.99	0.71–1.41	0.99			
T category (cT3-4)	1.43	1.04–1.93	0.02	1.68	1.82–2.34	0.008
N category (N0)	0.81	0.50–1.30	0.39			
M category (M0)	0.98	0.54–1.77	0.96			
Tumor size (≥ 5 cm)	0.86	0.58–1.26	0.44			
Vascular invasion (yes)	1.26	0.77–2.09	0.35			
Perineural invasion (yes)	0.77	0.49–1.20	0.26			

*CEA* carcinoembryonic antigen, *BMI* body mass index

*After univariate analysis, variables with a *P* value < 0.05 were entered into the multivariate analysis by a multiple logistic regression model

### Sexual and urinary function

The analysis of the questionnaires completed by 81% of patients who underwent robotic TME or APR. It shows that sexual function and general sexual satisfaction decreased significantly 1 month, 6 months, and 1 year after intervention, respectively. In male patients, the scores for erectile function were 18.8 ± 2.7 (preoperation) versus 12.1 ± 4.6 (*P *= 0.008) at 1 month and 14.2 ± 5.1 (*P* = 0.021) at 6 months; for general satisfaction, 6.7 ± 1.2 (preoperation) versus 5.1 ± 1.2 (*P* = 0.017) at 1 month and 5.2 ± 1.2 (*P* = 0.023) at 6 months. In female patients, the values for arousal were 2.5 ± 0.9 (preoperation) versus 0.8 ± 0.4 (*P* = 0.024) at 1 month and 1.9 ± 0.6 (*P* = 0.068) at 6 months for general satisfaction. Both parameters then increased progressively, and at 1 year after surgery, the values were comparable to those measured before surgery. These data are presented in Table 5.

**Table 5:** Sexual function data

IIEF	Range	Before surgery	30 days after surgery	6 months after surgery	1 year after surgery
Male					
Erectile function	(0–30)	18.8 ± 2.7	12.1 ± 4.6*	14.2 ± 5.1*	16.8 ± 5.1
Orgasmic function	(0–10)	6.3 ± 1.6	4.2 ± 1.8*	5.0 ± 1.9*	5.8 ± 1.7
Sexual desire	(2–10)	5.5 ± 1.2	4.5 ± 1.1*	5.1 ± 1.3	5.7 ± 1.4
Intercourse satisfaction	(0–15)	7.3 ± 2.0	4.4 ± 1.3*	5.6 ± 1.7*	6.9 ± 1.6
Overall satisfaction	(2–10)	6.7 ± 1.2	5.1 ± 1.2	5.2 ± 1.2*	5.6 ± 1.3
Erectile function	(0–30)	18.8 ± 3.3	12.1 ± 4.6*	14.2 ± 5.1*	16.8 ± 5.0
Orgasmic function	(0–10)	6.3 ± 1.6	4.2 ± 1.8*	5.0 ± 1.9*	5.8 ± 1.7
Female					
Desire	(1.2–6)	2.3 ± 0.6	1.8 ± 0.5*	2.1 ± 0.5	2.4 ± 0.6
Arousal	(0–6)	2.5 ± 0.9	0.8 ± 0.4*	1.9 ± 0.6	2.2 ± 0.8
Lubrication	(0–6)	2.0 ± 0.8	0.8 ± 0.3*	2.1 ± 0.5	2.3 ± 1.0
Orgasm	(0–6)	2.5 ± 1.0	0.8 ± 0.4*	2.2 ± 0.4	2.5 ± 1.1
Satisfaction	(0.8–6)	2.5 ± 0.9	0.7 ± 0.3*	1.8 ± 0.3	2.4 ± 0.9
Pain	(0–6)	2.2 ± 0.8	0.8 ± 0.4*	1.9 ± 0.3	2.5 ± 0.9

Data was expressed by Mean ± SD. The nonparametric Kruskal–Wallis test was employed to compare the qualitative variables

*IIEF* International Index of Erectile Function, *FSFI* Female Sexual Function Index

*P < 0.05

Concerning urinary function, the grade of incontinence measured 1 year after the intervention was statistically unchanged when compared with the preoperative status for both sexes. These data are summarized in Table 6. In particular, in male patients, we observed no significant deterioration of voiding or incontinence during the entire study period. Filling symptoms and incontinence function in women were both statistically worse 1 month after intervention, 2.3 ± 0.6 versus 3.2 ± 0.8 (*P* = 0.024) and 1.5 ± 0.6 versus 2.9 ± 0.8 (*P* = 0.019), respectively, whereas at 1 year after surgery, all scores were comparable to preoperative values. We observed the same results when comparing the total number of male and female patients with severe or moderate urinary incontinence (score ≥ 9) before and 1 year after surgery.

**Table 6:** Urinary function data

ICIQ-MLUTS	Range	Before Surgery	30 days after surgery	6 months after surgery	1 year after surgery
Male					
VS	(0–20)	3.5 ± 1.4	3.6 ± 1.2	2.7 ± 1.0	3.1 ± 1.1
IS	(0–20)	2.4 ± 0.9	2.6 ± 0.6	2.1 ± 0.6	2.3 ± 0.5
Female					
VS	(0–20)	2.4 ± 0.7	4.3 ± 1.1*	2.9 ± 0.9	2.3 ± 0.7
IS	(0–20)	1.5 ± 0.6	2.9 ± 0.8*	1.9 ± 0.6	1.8 ± 0.7
FS	(0–20)	2.3 ± 0.6	3.2 ± 0.8*	2.0 ± 0.7	2.4 ± 0.6

Data were expressed by Mean ± SD. The nonparametric Kruskal–Wallis test was employed to compare the qualitative variables

*ICIQ*-*MLUTS* International Consultation on Incontinence Male Lower Urinary Tract Symptoms questionnaires, *ICIQ*-*FLUTS* International Consultation on Incontinence Female Lower Urinary Tract Symptoms questionnaires, *VS* indicates voiding symptoms, *IS* incontinence symptoms, *FS* filling symptoms

*P < 0.05

With regards to the impact of urinary symptoms on patient’s QoL, no difference was measured in either sex at 1 year compared to preoperative status (Table 7). For example, the scores of voiding QoL were 3.7 ± 1.1 (preoperation) versus 3.3 ± 1.1 (1 year, *P* = 0.162) for male and 3.7 ± 0.9 (preoperation) versus 3.7 ± 1.0 (1 year, *P* = 0.955) for female.

**Table 7:** Impact on QoL of urinary function in patients

ICIQ-MLUTS	Range	Before surgery*	30 days after surgery*	6 months after surgery*	1 year after surgery*
Male					
V QoL	(0–20)	3.7 ± 1.1	5.3 ± 1.6	3.4 ± 1.0	3.3 ± 1.1
I QoL	(0–20)	2.2 ± 0.6	1.2 ± 0.6	1.5 ± 0.6	2.2 ± 0.5
Female					
V QoL	(0–20)	3.7 ± 0.9	5.3 ± 1.5*	3.6 ± 1.1	3.7 ± 1.0
I QoL	(0–20)	1.4 ± 0.6	2.9 ± 0.8*	1.8 ± 0.6	1.6 ± 0.7
F QoL	(0–20)	5.2 ± 1.6	7.6 ± 1.8*	6.5 ± 1.7	5.6 ± 1.6

Data were expressed by Mean ± SD. The nonparametric Kruskal–Wallis test was employed to compare the qualitative variables

*V QoL* indicates voiding quality of life, *I QoL* incontinence quality of life, *F QoL* filling quality of life

*P < 0.05

### Long-term oncological outcomes

The median follow-up duration from primary treatment was 34.6 months (25th–75th percentile 18–79). A total 1034 of 1145 patients underwent R0 resection. Local recurrence and distant metastases occurred in 24 (2.3%) and 218 (21.1%) patients, respectively. The 3-year disease free-survival (DFS) rate was 81.0%, and the 3-year overall survival (OS) rate was 87.2%.

## Discussion

We reported here the results of a large consecutive nonselected number of robotic rectal resections from real world to evaluate the safety and efficacy of robotic procedure for rectal cancer in China. In this study, we contributed to followed data analysis: (1) we analyzed the patients’ short-term outcomes and identified high-risk patients for robotic rectal surgery. (2) We summarized the sexual and urinary function data, as well as the long-term oncologic outcomes in detail. All this data demonstrated that the robotic procedure was safe and efficacious for patients with rectal cancer, when performed in an experienced medical unit.

This study summarized the complication and identified the risk factor of surgical complications during RRR. The incidence of grade 3 and grade 4 complications together was 2.4% in this RRR study, which was lower than rates reported in previous laparoscopic rectal resection (LRR) studies of 7.4% [22] and 9.5% [23]. A systematic review demonstrated no differences in postoperative morbidity and mortality between LRR and ORR [2]. RRR may help to reduce severe surgical complications; therefore, we recommended patients at a high risk for surgical complications undergo robotic surgery for rectal cancer.

More importantly, by applying a standardized robotic approach as described, we effectively reduced the incidence of anastomotic leakage (totally 4.2%) without including a routine diverting stoma. The rate of AL was 0.8% for AR and 6.6% for LAR, which was obviously lower than the reported 10–13% in recent studies [6, 8, 20]. It is worth noting that previous studies, even multicenter RCT studies, had low operative numbers performed by a single doctor; for example, 30 units completed 739 cases of LRR in the COLOR II study [8] and 40 surgeons completed 237 cases of RRR in the ROLARR study [6]. Limited case volume and inadequate surgical experience may compromise the quality of surgery [24, 25], which may be a potential reason possibly responsible for the high rate of AL in previous studies.

Independent risk factors for surgical complications during RRR were male gender, tumors located at the mid-low rectum, combined organ resection, and clinical T category (cT3–4). Risk factors previously reported in LRR or ORR [22, 26] studies, BMI ≥ 28, age ≥ 75, history of comorbidity, preoperative radiotherapy (RT) or chemoradiotherapy (CRT), and tumor size ≥ 5 cm, were not significant in our robotic procedure. These differences may be due to the diversity of patient characteristics: (1) The neoadjuvant RT or CRT was associated with an increased rate of anastomotic leakage (AL) as reported previously [26]. In this study, only patients with T4 or N2 disease received preoperative RT or CRT, and other patients with a high risk of relapse received chemoradiotherapy after radical surgery. The number of patients with mid-low rectal cancer who received neoadjuvant RT or CRT was 23.7% (181/778) in this study, unlike a greater proportion of CRT (46–58%) in Western countries [6, 8]. (2) Regarding a history of comorbidities, this study only collected data from cardiovascular disease and diabetes; therefore, some data associated with other organ systems were missed. (3) The percentage of patients with a BMI ≥ 28 was about 18% in this study. BMI was significantly lower in Asian people than the patients in western who have a high proportion of obese patients. [27, 28].

This study supplied larger cases analysis by questionnaires to evaluate the role of robotic TME on preservation of urogenital function. We recorded no difference in terms of incontinence, filling, or voiding symptoms at 1 year after surgery compared to the preoperative status for both sexes. This result was similar to that of a previous robotic study [10] and better than that of a LRR study, which was associated with a rate of sexual and urinary dysfunction higher [8] or comparable [29] to that following open surgery. Limitations of LRR can be explained by the technical complexities of laparoscopy surgery, including the unstable view of the operative field and the poor ergonomics of the surgical tools that render complex operations more difficult with a higher degree of surgeon fatigue. However, robotic surgery can overcome the limitations of laparoscopic surgery for rectal cancer, offering the surgeon a stable camera platform with a 3-dimensional operative field, precise and dexterous control of the wristed instruments to improve endo-wrist function, and reduced operator fatigue. As reported previously [30], the technical characteristics of a robotic system permits extended lymph node dissection and an accurate dissection of the smaller anatomical structures, thereby protecting the pelvic autonomic nerves.

In terms of the oncologic aspects, we found the 3-years DFS and OS were 81.0% and 87.2%, respectively, which was comparable to recent reports of LRR and ORR results [23, 31]. This outcome can be interpreted as confirmation that the therapeutic effectiveness of RRR is equivalent to laparoscopic and open surgery. Interestingly, the local recurrence rate and positive circumferential margin rate were better than that of LRR [7, 23] and ORR [31] in previous studies. Lim et al. [7] reported a lower local recurrence rate in RRR (2.7%) than that of LRR (6.3%) for patients with mid-low rectal cancer following neoadjuvant chemoradiation therapy. Baek et al. [32] classified 182 patients who underwent robotic surgery for rectal cancer into easy, moderate, and difficult groups by MRI-based pelvimetry and there was no difference between the groups in terms of operative and pathologic outcomes. Thus, an advantage of RRR may be its ability to overcome challenges associated with difficult pelvic anatomy and allow for a high quality of tumor resection.

The current study has several limitations in that it is single-arm large cases analysis with data from only a single center and is not a head-to-head designed study.

## Conclusion

Based on the results, we determined robotic rectal resection to be a safe and adequate technique for the treatment of rectal cancer, with a low incidence of serious complications and anastomotic leakage. Robotic TME allows for preservation of urinary and sexual functions in patients with mid-low rectal cancer.
